# Factors Affecting Quality of Life and Longevity in the Elderly People in Phrae City, Thailand

**DOI:** 10.31372/20200502.1081

**Published:** 2020

**Authors:** Laddawan Daengthern, Somsak Thojampa, Kittisak Kumpeera, Chommanard Wannapornsiri, Roongtiva Boonpracom

**Affiliations:** Naresuan University, Phitsanulok, Thailand

**Keywords:** empowerment, elderly people, quality of life, longevity

## Abstract

The purpose of this descriptive research was to study the factors that influence the quality of life (QOL) and longevity of the elderly in Thailand. The sample was made up of 280 elderly people in Phrae province, Thailand. The research found that (1) the QOL of the elderly in Phrae province was overall at a good level of 66.30%, (2) the factors that have significant influence on the QOL of the elderly at the *p* value = .01 are healing and exercise factors which are able to jointly predict the QOL of the elderly in Phrae Province by 12.2% (*R*^2^ = .122), and (3) from the structured interviews of 10 elderly people aged 80 years and older, it was found that diet, exercise, stress reduction, and healing are factors that allow the elderly to live for an average of 80 years which is above the average age of Thai people (the average age for men is 71.8 years old and for women is 78.6 years old).

## Introduction

The National Statistical Office (2018) conducted a survey of Thai people around the country in 2017 and identified the overall number of elderly people, those at the age of 60 years old and older, in the country is at 10,225,322. Among them, 2,093,071 elderly people, who accounted for 15.45% of the total number, lived in the North of Thailand. Nong Muang Khai District, Phrae City is located in Northern part of Thailand, the population is 17,803 people, comprising of 8,376 men and 9,427 women. Tamnak Tham Sub-district, which is in Nong Muang Khai District, has an approximate total area of 11,125 km^2^. The total population of Tamnak Tham Sub-district is 3,336 people of which 845 are elderly people (25.32%) divided into 350 men and 495 women (Tamnak Tham Development Project Report (2017). It can be seen that Tamnak Tham Sub-district has more than 20% elderly people in their population which means that the sub-district is a complete ageing society (Anantakun, 2017) therefore this part of Phrae city was chosen to be the setting of this study. In addition, knowledge of healthcare, including effective technology, makes the age of the population more than the expected average age of Thai people which is 71.8 years old for men and 78.6 years old for women. The average number of years that people over the age of 60 will continue to live is expected to be 20.1 years for men and 23.4 years for women. Furthermore, the average number of years that people over the age of 65 will continue to live is expected to be 16.4 years for men and 19.3 years for women (Institute for Population and Social Research, Mahidol University, 2016).

According to the Institute for Population and Social Research, Mahidol University the population is more likely to live to 100 years’ old which is more than in the past due to advances in medicine and public health (Institute for Population and Social Research, Mahidol University, 2016). It is not difficult to achieve this nowadays because Thai people have knowledge on how to take care of themselves and how to take better care of their health. Elderly Thai people want to live longer so they avoid doing risky things and are retaining in their minds to be stricter on correct food intake by not using much oil, frying, and not using coconut milk. All of which increase health, longevity, and quality of life (QOL) throughout their lives. In less than 20 years, Thailand will be a complete ageing society, which will have 14 million elderly people or 20% of the country’s population (Kiatsawee, 2013).

Promoting the present and future of the elderly to be self-reliant in the long run as much as possible is done by looking after their physical, mental, emotional, and social health so that they can live alone but happily. They can also learn to use technology so that they can live alone and happily with themselves and their surrounding environment by being able to communicate closely with family members even when staying away through the use of communication technology (Social Media) such as mobile phones, computers, Internet, social networks such as Facebook, Line, Twitter. This is in order for the elderly to be able to catch up with the modern world into society and become closer to their children and friends (Srisathitnarakun, 2010). With the common goal of supporting the elderly to love happily until the end of their lives, which has been taken care of by families and communities to have a good QOL, long life, and most importantly, be self-reliant as long as possible (Srisathitnarakun, 2010).

QOL means that the individual’s perception of life status under cultural context and their value system in relation to goals, expectations, standards and concerns, is living with complete health and stability which includes physical, emotional state, social, and spiritual well-being (World Health Organization (WHO), 1996). In addition, the WHO (1996) has summarized the four components of QOL as follows: physical, mental, social relations, and environmental. Mahatnirunkul, Sinpakij, and Poomphaisanchai (1998) developed a measure of QOL and widely used in research studies in Thailand on the QOL of general people and the elderly.

For the purpose of this study, QOL is operationalized as an individual’s perception of life status under cultural context and their value system in relation to goals, expectations, standards, and concerns, is living with complete health and stability which covers physical, emotional state, social, and spiritual well-being (WHO, 1996).

Because QOL is an important goal and is something that people want in their own lives, having a good QOL will show that a person has well-being, is healthy, strong, and can live a normal life in society (Chamsaeng, Kensila, Tangchaidee, & Kongsomboon, 2012). In order for the elderly to have a good QOL, good physical health, can live in society, and have a long life, Kiatsawee (2013) discussed the following four factors:

Food is considered to be a big issue in daily life. Research was conducted by interviewing seniors aged 80 years and over in Thailand and found that most of the food that they eat are natural foods such as homegrown vegetables or fruits, fish that are obtained from natural water sources in the community, and eating three full meals daily, especially breakfast and lunch (Krachanhchom & Champawan, 2018; Sapma & Sakdiworaphong, 2014).

Exercise is the movement of the body that helps the muscle to not get stuck, helps in good blood circulation, and making it easier to walk. In addition, exercise helps to meet more people, talk, socialize, reduce nervous symptoms, and nervousness to make the mind clear which proper exercise will help in increasing longevity (Krachanhchom & Champawan, 2018).

Stress reduction is an important factor related to body and mind. Consistent with the research of Krachanhchom and Champawan (2018), they found that the elderly aged 80 years and older who live close to nature, enjoy the cultivation of vegetables, living together as a large family, and eating with children can affect mental health and reduce stress.

Treatment is the access to public health services and is a very important factor including medical and public health advancement. When there are health problems, they get medical advice, follow the doctor’s instructions, take prescription drugs, and follow-up appointments with the doctor. All of these will cure the disease that can be treated, and it will reduce suffering if diseases are incurable (Krachanhchom & Champawan, 2018).

There are four QOL domains:

Physical domain means perception of the physical condition of the elderly which affects daily life such as the recognition of the condition of physical well-being, perception of being comfortable, no pain, awareness of strength in daily life, the awareness of independence that does not depend on others, awareness of one’s ability to move their body, awareness of their ability to carry out their daily activities, recognition of ability to do work, recognizing that one does not have to rely on drugs or other medical treatment.

Psychological domain means the awareness of one’s own mental state such as perception of feelings, thoughts, memory, concentration, decisions and the ability to learn from various life stories of one’s self-awareness, of being able to manage sadness or anxiety, awareness of their various beliefs that affect the lifestyle such as awareness of spiritual beliefs, religion, meaning of life, and other beliefs that has a positive effect on life to overcome obstacles.

Social relationships domain mean the awareness of one’s relationship with other people, awareness of receiving help from others in society, acknowledgment that one is aiding other people in the society, and including the awareness of sexual emotions or sexual intercourse.

Environment domain means awareness about the environment that affects the way of life, such as the perception that they live independently, not imprisoned, safe and secure in their lives, perception of being in a good physical environment without various pollution, have convenient transportation, with financial resources available, health services, and social welfare center. It is also a perception that they have the opportunity to receive news or practice various skills, perception of having recreational activities, and free time activities

There is currently no study in the public domain on the quality of the elderly in Phrae City, Thailand. Studying the factors that influence the QOL and longevity of the elderly is most important in Thailand since it is projected to be a complete ageing society in 20 years (Kiatsawee, 2013). Therefore, the researchers were interested in studying the factors that help the elderly in Phrae City have a good QOL and longevity and to find the relevant factors and analyze them to develop empowerment programs for the elderly to become more involved in their self-care. Self-reliance creates confidence in their ability to control diseases and can choose the right way to take care of themselves.

### Research Objectives

To study the factors such as food, exercise, stress reduction, and treatment that influence the QOL and longevity of the elderly in Phrae City, Thailand.

## Methodology

This was a descriptive study conducted by surveying the factors that influence QOL and longevity of the elderly. Data were collected between August and September 2019.

### Population and Sample

The sample included elderly people living in Tamnak Tham Sub-district, Nong Muang Khai District, Phrae City, Thailand who were willing to participate in the research. The 272 participants size was calculated using the formula of Taro Yamane (Kiatsawee, 2013). Inclusion criteria included: (1) aged 60 years old and above, (2) is an elderly living in a domicile in Tamnak Tham Sub-district, Nong Muang Khai District, Phrae City, Thailand, (3) able to communicate by speaking, reading and writing Thai language, and (4) willing to cooperate in the research projects. Exclusion criteria included: (1) have a serious disease or cannot complete activities of daily living independently and (2) cannot help themselves, such as patients who are bedridden, must rely on others to help communicate, or cannot participate in the research projects.

### Instruments

The instruments consisted of the questionnaire on the factors affecting QOL and longevity by Kiatsawee (2013) which consists of four factors namely food, exercise, stress reduction, and treatment factors with 30 items, and the questionnaire with 26 items on the QOL by the WHO (1996) with four components which are physical, psychological, social relations, and environment which was developed from Mahatnirunkul et al. (1998). This new questionnaire was developed to adapt to the culture of the sample which is different from the sample where the original questionnaire was used. The QOL questionnaire was rated by using a 5-point Likert scale, 1 being the lowest and 5 being the highest score. The total score of the responses in this questionnaire is 130. The QOL score was computed by getting the mean and was described into three levels namely: poor (26–60), moderate (61–95), and good (96–130).

#### Evaluation of Instrument Quality

The researchers evaluated the quality of the questionnaire as follows:

Content validity was done by sending the questionnaires and the structured interview form in four factors to three experienced experts to check. After the experts gave scores for each item of the questionnaire, the researchers analyzed the content validity. It was found that the questions had a consistency index between 0.6 and 1.0.

Reliability was done after checking the content validity and editing the questionnaire, then the questionnaire was tested using a try-out. The try-out was conducted with the elderly aged 60 years and over who have characteristics similar to the sample of 30 elderly people. After that, the results were tested and analyzed for the reliability of the questionnaire by calculating the Cronbach’s alpha coefficient; part 2 and 3 were 0.95 and 0.88, respectively.

### Rights Protection for Sample

This study was approved by the Human Ethics Committee of Naresuan University (COA No. 355/2019, IRB No. 0105/62) 7 August 2019 to 7 August 2020.

### Data Collection

The researchers requested permission to collect data from the president of Tamnak Tham Sub-district Administrative Organization for the data of the elderly.

The researchers coordinated with the community developers who are the coordinators of Tamnak Tham Sub-district to clarify the purpose of the research, the benefits of the research, including the protection of sample rights and data collection methods.

The researchers performed data collection.

(3.1) The data were collected by the researchers by interviewing 10 elderly people who are 80 years old or older by choosing specifically 1–2 people each from the eight villages of Tamnak Tham Sub-district, Nong Muang Khai District, Phrae City.

(3.2) The researchers assigned the coordinators of Tamnak Tham Sub-district Organization to distribute the questionnaires to a sample of 272 elderly people. It took 4 weeks for the data collection to be completed. The researchers received back 270 completed questionnaires, which was 99.26% of the sample.

### Data Analysis

This research analyzed data using univariate statistics: frequency, percentage, mean, standard deviation, and stepwise multiple regression analysis. All tests were carried out at an alpha level of significance of 0.05. All data were analyzed using IBM SPSS Statistics.

## Results

### Demographics

A total of 270 participants were recruited for this study and all of them completed the questionnaire. The majority of participants were females (63%). The average age was 70.91 years old. Most of the participants were between 60 and 70 years old (48.15%) followed by 70–79 years old (37.41%). Majority of the sample were married (49.26%) followed by those who were widowed/divorced/separated (44.44%). Most of them had primary education (76.30%) and were retired (61.85%) which was followed by farmers (21.48%). The average monthly income was 1,882.56 Baht (62 USD) which is not enough for daily living. 57% of the sample have underlying illness while 73.70% have chronic diseases that requires continuous medication such as hyperlipidemia, hypertension, and diabetes mellitus. Most of the elderly do not smoke (84.44%) and do not drink alcohol (71.85%).

### Level of Factors of QOL

The level of QOL was computed by obtaining the mean of the score of the responses from the questionnaire. This was divided into three levels namely: poor (*x¯*: 1–2.33), moderate (*x¯*: 2.34–3.67), and good (*x¯*: 3.68–5), which were adapted from the original tool. The elderly people in Tamnak Tham Sub-district, Phrae City have a good level of QOL (*x¯* = 3.99, SD = 0.45). When each factor is considered, it was found that the treatment factor had the highest mean, followed by stress reduction, exercise, and food factors, respectively. The level of factors related to the QOL of the participants are shown in Table 1.

**Table 1:** Mean, Standard Deviation, and the Level of Factors related to the Quality of Life of the Elderly in Tamnak Tham Sub-district, Phrae City, Classified by Each Factor and Overall (N = 270)

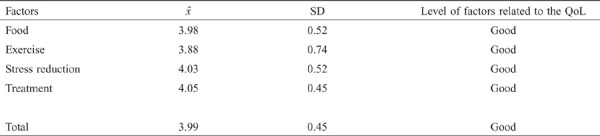

### Domains of QOL

The elderly in Tamnak Tham Sub-district, Phrae City have an overall QOL at a good level with 66.30%. When considered in each factor, it was found that the social relations and environment domains were at a good level, while the physical and psychological domains were at a moderate level, as shown in Table 2.

**Table 2:** Levels of the Quality of Life of the Elderly in Tamnak Tham Sub-district, Phrae City, Classified by Each Domain and Overall (N = 270)

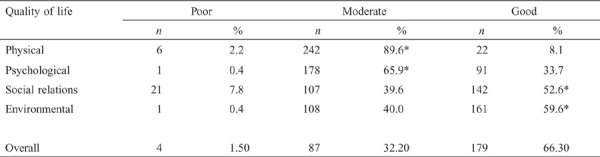

### Predictors of QOL

The predictors with the highest standard score were the treatment factor (beta = .215) followed by the exercise factor (beta = .208), respectively. It shows that the treatment factor is the first priority followed by exercise factor in predicting the good QOL of the elderly in Tamnak Tham Sub-district, Phrae City which is 12.2% (*p* < .01) shown in Table 3.

**Table 3:** Regression Coefficient in Raw Scores (b), Beta, and the Test of the Significance of the Multiple Regression Coefficients used to Predict Quality of Life and Longevity of Elderly in Tamnak Tham Sub-district, Phrae City (N = 270)

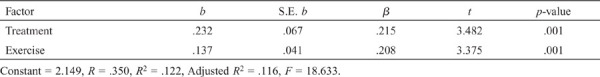

## Discussion

There were 270 participants recruited in this study. This present study demonstrated that the factors affecting the QOL and longevity of the elderly in Tamnak Tham Sub-district, Phrae City can jointly predict the QOL and longevity of elderly people with a statistical significance.

(1) The QOL of the elderly people in Tamnak Tham Sub-district, Phrae City was at a good level of 60.30% overall. When considering each factor, it was found that social relationships and environmental factors were at a good level, physical and mental factors are at a moderate level, and this explains that the social relationships, environmental, physical, and mental factors were important for the QOL and those are important thing that people want to achieve in their own lives. Having a good QOL shows that a person has a good life, good physical health, and can live a normal life in the society (Chamsaeng et al., 2012). This is also consistent with the research of Ngammeerith, Rarukchati, and Phowan (2018) who studied the QOL in the central region of Thailand. They found that the QOL of the elderly people there was at a good level. When considering each item in the questionnaire, the elderly in Tamnak Tham Sub-district has scores reflecting a good life satisfaction, have the ability to perform daily activities well, sleep well, have peace of mind, and have a good environment including receiving care from health facilities at a high to a very high level.

QOL is achieved by being able to live happily and peacefully under the context of society, culture, and values that people have including being able to adjust themselves with the society and the environment happily and by making others happy depending on their personal satisfaction in life, their satisfaction of their living conditions, and acceptance of their own hardships (Phiansrivajara & Paramatthakon, 2010). The elderly in Tamnak Tham Sub-district received good health promotion for good health and stability which included four factors; physical, emotional, social and environmental, and spiritual health. This was with cooperation from all sectors of the Tambon Administrative Organization of Tamnak Tham Sub-district, Office of Social Development and Human Security, and the Phrae City Hospital. They participated in organizing additional activities for the elderly people such as vocational training for women by weaving bags from plastic strands for selling. The elderly were also able to earn extra income from the sales of the products.

In addition, an elderly club was established at the district level and a school was established for the elderly by the Office of Social Development and Human Security Phrae City. This elderly school conducts activities to provide knowledge about health and exercise for the elderly people (Tamnak Tham Development Project Report, 2017). From the interviews with the elderly, it was found that there were exercises that were practiced consistently and done together in the elderly school such as the Ramwong Khorngka dance with three songs that take approximately 10 to 15 minutes every Wednesday. In addition, if the elderly were stressed, they talked with each other and went to school every Wednesday to relieve their stress. They also made various products every Monday, Wednesday, and Friday. These activities resulted in improving the health of the elderly by developing the potential of health self-care with exercise and making various products. The researchers believe that health promotion in the elderly will help to improve their QOL.

(2) The factors affecting the QOL and longevity of the elderly in Tamnak Tham Sub-district, Phrae City which can jointly predict with a statistical significance of .01 are the treatment factor which is of primary importance in predicting the good QOL of the elderly and followed by exercise factor. Both factors can predict the QOL of the elderly in Tamnak Tham Sub-district by 12.2%. This is consistent with the research of Drewnowski and Evans (2001) which studied the diet, exercise, and good QOL of the elderly; it found that the exercise has a statistically significant relationship for the long life of the elderly. In addition, the research of Phiansrivajara and Paramatthakon (2010) found that exercise of the elderly results in good health, including paying attention to self-care by having a health check annually. Furthermore, the quality of public health and medical services also increase the QOL and longevity of the elderly. The elderly in Tamnak Tham Sub-district with chronic illnesses who needed to be cared for with public health services are at 73.70%.

From the interviews, it was found that the treatment factor occurred when the elderly regularly took medication to treat their chronic illnesses. Some people forget to take their medication but there are appropriate management methods to solve this problem, and the elderly with chronic illnesses goes to the doctor regularly for follow up. Kiatsawee (2013) mentioned that access to public health services is a very important factor. This is consistent with each item in the questionnaire which found that the elderly in Tamnak Tham Sub-district, Phrae City can access public health services as needed at a high level. When there are health problems, they meet and consult a doctor early and strictly follow the advice of doctors such as taking medications, follow up consultation with the doctor, follow all the restrictions recommended by the doctor which causes illnesses to subside, diseases that can be cured will disappear but if it is a disease that cannot be cured then they can alleviate their suffering.

For the exercise factor, Kiatsawee (2013) and Siriphanich (2007) mentioned that walking for at least 30 to 60 minutes a day to work or doing activities in daily life, or walking for at least 2.5 hours a week will increase the life of up to 7 years and brisk walking for 75 minutes per week can increase the life for up to 2 years. In addition, Siriphanich (2007) and Drewnowski and Evans (2001) mentioned that with exercise people can socialize, reduce nervous system disorders and worries, and make the mind clear. The appropriate exercise will help in longevity, as in the interviews with the elderly aged 80 years and over which found that food, exercise, stress reduction, and treatment factors are important for the elderly to live longer to help themselves.

Regular exercises are done together with friends in the elderly school by doing the Ramwong Khorngka dance with three songs every Wednesday that takes 10–15 minutes, and the elderly exercise at home by walking every day for about 30 minutes by themselves. This is same as the study of Krachanhchom and Champawan (2018) which is entitled “The study of food and consumption that leads to longevity of the elderly in Chartiphan Thai-Yuan group, Mae Chaem District, Chiang Mai City,” the sample consisted of eight seniors aged 80 years and older by using structured interviews. This study found similar characteristics to the elderly in Tamnak Tham Sub-district in walking, exercising 20–30 minutes per day for 3–4 days a week, and eating natural food. Chili sauce is one of the food items in each meal which is eaten with raw vegetables or steamed vegetables.

As for the social relationship and environment factors, most of the elderly were satisfied with their life, such as religious practices and having good relationships with their children and neighbors. They have good resting places, good ventilation, and public utility systems are at a good level, with quick and convenient access to public health services. This includes how the elderly and members of the family pay attention and follow the culture and traditions in the community that have been passed on continuously and strictly (Krachanhchom & Champawan, 2018).

## Conclusion

In conclusion, we found that social relationships and environmental factors were at a good level, physical and mental factors were at a moderate level. This may explain why the social relationships, environmental, physical, and mental factors were important for the QOL. The research result can be considered for intervention research for the elderly to have good QOL and longevity.

## Recommendations

Since the physical and psychological domains of QOL of the elderly people in Tamnak Tham Sub-district, Phrae City were at a moderate level, an empowerment program to improve their physical and psychological needs could be implemented to increase their QOL.

## Acknowledgments

The researchers would like to thank the elderly at Phrae City, Thailand for agreeing to be the sample group. We would also like to thank all the experts for checking, correcting, and providing useful suggestions for effective research tools. Also, we would like to thank the Thai Health Promotion Foundation for supporting this research.

## Declaration of Conflicting Interests

The authors declared no potential conflicts of interest with respect to the research, authorship, and/or publication of this article.
